# Assessment of Polarimetric SAR Interferometry for Improving Ship Classification based on Simulated Data

**DOI:** 10.3390/s8127715

**Published:** 2008-12-02

**Authors:** Gerard Margarit, Jordi J. Mallorqui

**Affiliations:** 1 Remote Sensing Laboratory, UPC, C Jordi Girona, 1-3, Campus Nord, E-08034, Barcelona, Spain E-mail: mallorqui@tsc.upc.edu.; 2 GMV Aerospace and Defense, S.A., C Balmes, 268-270, 5th floor, E-08006, Barcelona, Spain

**Keywords:** POLInSAR, Ship Classification, SAR Simulation, Coherent Target Decompositions

## Abstract

This paper uses a complete and realistic SAR simulation processing chain, GRECOSAR, to study the potentialities of Polarimetric SAR Interferometry (POLInSAR) in the development of new classification methods for ships. Its high processing efficiency and scenario flexibility have allowed to develop exhaustive scattering studies. The results have revealed, first, vessels' geometries can be described by specific combinations of Permanent Polarimetric Scatterers (PePS) and, second, each type of vessel could be characterized by a particular spatial and polarimetric distribution of PePS. Such properties have been recently exploited to propose a new Vessel Classification Algorithm (VCA) working with POLInSAR data, which, according to several simulation tests, may provide promising performance in real scenarios. Along the paper, explanation of the main steps summarizing the whole research activity carried out with ships and GRECOSAR are provided as well as examples of the main results and VCA validation tests. Special attention will be devoted to the new improvements achieved, which are related to simulations processing a new and highly realistic sea surface model. The paper will show that, for POLInSAR data with fine resolution, VCA can help to classify ships with notable robustness under diverse and adverse observation conditions.

## 1. Introduction

The GRaphical Electromagnetic COmputing SAR (GRECOSAR) simulation tool has been developed at UPC [1, 2]. The possibility to process any complex geometry within flexible and totally controlled scenarios has provided a new benchmark for carrying out a set of SAR research activities that are difficult to be done with real data. Examples are exhaustive scattering studies where the dispersion behavior of targets is evaluated for the widest range of observation conditions possible or performance tests where current and/or new sensor designs are evaluated according to particular specifications.

GRECOSAR has been designed to deal with any type of SAR sensor operating at any band, resolution and mode with polarimetric and interferometric capabilities. Its processing efficiency is high and only needs from a simple PC to process realistic and highly detailed 3D models. Up to now, two main types of scenarios with dielectric materials can be managed: maritime and urban. The former is very realistic as ship motions, bearing and velocity are considered as well as a surrounding dynamic sea.

GRECOSAR has been mainly used for scattering studies of complex targets. Most of the efforts have been focused to ships for which few information about their dispersion characteristics was available. This is essential for developing more efficient scattering-based classification methods that can fulfill the new monitoring demands for SAR imagery (see IMPAST [3] and DECLIMS [4] projects). In this framework, a large number of polarimetric scattering maps with resolutions around centimeters have been generated for different ship geometries and environmental parameters. Their analysis with Coherent Target Decompositions (CTD) [5, 6] have revealed certain dispersion stability along the radar aspect angle. The cause is the presence of Permanent Polarimetric Scatterers (PePS) that have a high Radar Cross Section (RCS) and a well-defined scattering pattern, which remains constant within a large solid angle [7, 8]. The spatial and polarimetric distribution of PePS has appeared to be particular for each vessel model, characterizing its macro-scale features. This has allowed the definition of a *feature set* from which a new quantitative manner for ship discrimination is possible [7].

The feature set has been exploited to propose a new Vessel Classification Algorithm (VCA) that takes profit of the information diversity provided by POLInSAR and the specifications of the new generation of orbital sensors. A *similarity parameter* (S) is used to evaluate the correlation among the feature set estimated from SAR images and the reference ones defined from simulated imagery. In comparison to other classification approaches [9, 10], the decision rule is simpler and more robust as *only* one condition has to be evaluated, the value of similarity.

In the paper, all the details explaining the complete SAR simulation chain built with GRECOSAR and the resulting ship classification studies are presented. The paper compiles and extends the main conclusions obtained giving a comprehensive overview of what is disseminated in different papers. The main goal is to present how a complete numerical tool can help to make improvements in SAR image post-processing and, particularly, in ship classification.

## 2. GRECOSAR

GRECOSAR is a numerical tool capable to reproduce in simple PCs the SAR signatures of complex targets that orbital or airborne SAR sensors would provide in real scenarios [1][2]. It is based on the UPC's GRaphical Electromagnetic COmputing (GRECO^®^) solver [11] that estimates, for each single frequency, the RCS of 3D targets via high-frequency methods. Exhaustive tests performed with canonical and complex targets have validated the code [2, 11].

### 2.1. Overall description

Electromagnetic (EM) calculations are performed in GRECOSAR via a graphic-based approach for which a bitmap resident in the RAM memory is generated from the input model. By using a particular illumination point of view fixed by the user-defined Line of Sight (LOS) direction, GRECOSAR renders the model with the PC graphic card and isolates the visible entities (edges and surfaces) from the back-facing ones. Over these entities, EM methods are applied making RCS prediction faster and independent of the input geometry. The main EM methods used by GRECOSAR are:

- Physical Optics (PO) for perfectly conducting surfaces.
- Method of Equivalent Currents (MEC) with Ufimtsev's Physical Theory of Diffraction (PTD) coefficients or Mitzner's Incremental Length Diffraction Coefficients (ILDC) for perfectly conducting edges.
- Multiple reflection analysis by a Geometrical Optics (GO) + PO ray-tracing algorithm. Bi-static GO is used for all reflections except the last one, for which PO is used. GO divergence factors for curved surfaces are computed approximately.

All these methods have shown accurate RCS estimation performance according to several tests done in anechoic chambers and comparison with other codes [11,12]. In practical terms, they allow to analyze targets of electrical size as large as 2*^n^λ*/16, with a maximum phase error of *λ*/8, where *n* is the number of bits in which the distance to the observer is discretized. This means that, with a 24-bit discretization, targets as large as 10^6^*λ* can be managed with *λ* being the operating wavelength.

For a proper GRECO^®^ performance, input models should be modelled with parametric surfaces by using CAD tools. In this work, the CAD package GiD^®^ of the International Center of Numerical Methods for Engineering [13] has been adopted. Once defined, target models need from an additional tessellation meshing procedure that discretizes parametric surfaces into small planar facets. Otherwise, the PC's graphic card would not be able to deal with target's geometry.

Regarding the simulated scenario, any type of POLInSAR sensor can be defined for any desired imaging geometry. For maritime environments, ship bearing, motions and velocity are managed with a surrounding sea surface, which is updated by a user-defined dynamic pattern [8]. Due to GRECO^®^ restrictions, the sea is modeled with small facets* taking the dielectric properties of salt water into account. A complex relative permittivity of ∈ = 75 − *j* · 27 has been selected for a salinity of 35 practical salinity units (psu) and a temperature of 25°*C* [14, 15]. The vertical coordinate of each facet point within the sea (not the ship) is updated at each synthetic aperture position by a dynamic height profile, which is based on the two-scale model adopted in sea height estimation theory [16, 17]. Such option has been selected as better accommodates to the facet-based structure of input models as directly provides the values of the vertical z coordinate. This does not happen for sea spectrum theory [18, 19] where an intermediate bilinear interpolation step, which can increase processing time according to surface dimensions and discretizing accuracy, is required [8]. However, this option permits to easily introduce accurate wind effects on sea scattering and, for this reason, it will be evaluated to be included in GRECOSAR. By now, wind influence is simply tackled by only managing wind orientation.

**Figure 1.**
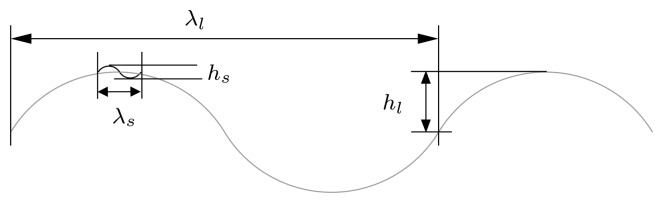
Profile of the two-scale sea surface model.

**Figure 2.**
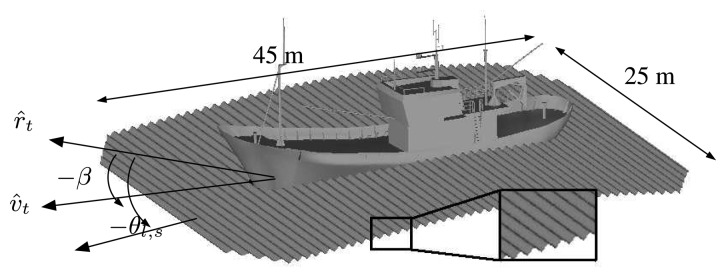
Snapshot of the 3D sea surface model. Note the small scale waves embedded over the surface of the large scale ones.

For sake of simplicity, a simple version of the two-scale approach has been used for which a small scale wave modulates the surface of a large scale one along time *t* by (see Fig. 1),

(1) $$h(x,y,t)=h_{l}\cos\{\kappa_{l}x^{\prime }-\sqrt{g\kappa_{l}}t\}+h_{s}\cos\{\kappa_{s}x^{\prime }-\sqrt{g\kappa_{s}}t\}$$

where surface dynamics are fixed by $\sqrt{g\kappa_{i}}$ with *g* being the acceleration of gravity and *κ_i_* = *2π*/*λ_i_* the wavenumber vectors of the larger (*i* = *l*) and smaller (*i* = *s*) scale waves. *x*′ = '*x*, *y*]^†^ is the transpose vector of the original set accomplishing *κ_l_x*′ = |*κ_l_*| · |*x*′| cos *θ_l_* with *θ_l_* being the wave course. At this point, GRECOSAR provides the chance to define the wave course of small scale waves, which may be different from *θ_l_*, according to the user-defined wind orientation. This permits to simply model wind effects without taking neither wind speed nor hydrodynamic forces into account. In future works, this drawback will be overcome by integrating into GRECOSAR more complete ways of wind simulation based on the spectral theory. The result of all this scenario simulation process lies on the maritime scenario of Fig. 2 where a correct simulation of sea-ship interaction is achieved [20].

Note that more advanced versions of the previous model have been developed to accurately retrieve physical parameters [14, 16, 17]. They do not normally assume one small scale wave, but an ensemble spanning with different wavenumber vectors [16]. In such a case, a mean *κ_s_* vector is defined and the small scale height *h_s_* is substituted by an integral that adds for each generic location the height contribution of all the small scale waves. Wind conditions are included via hydrodynamic theory. For the particular case of the current work, the model of Equation 1 is, however, enough as focus on evaluating the impact of sea clutter in SAR post-processing [21].

Sea model accuracy has been tested by comparing the Probability Density Function (PDF) derived from the clutter synthesized by GRECOSAR with the typical distributions associated with the scattering properties of real sea (Rayleigh for steady seas [22] and K distribution for swelled seas [23]). The results show that realistic clutter is achieved if facet dimensions are lower than λ/4 [8].

### 2.2. Main Simulation Steps

**Figure 3.**
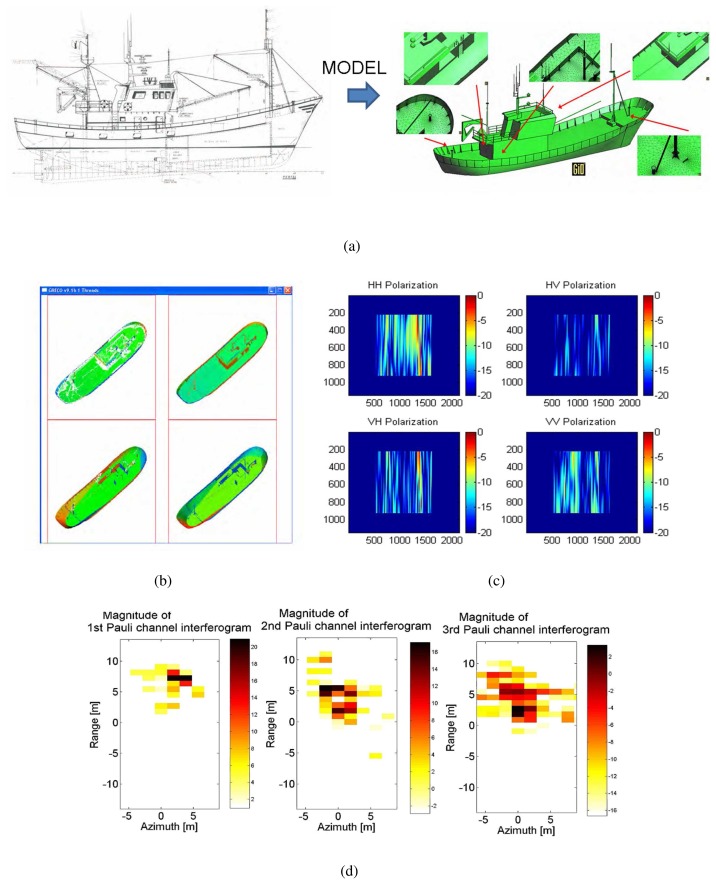
Series of snapshots providing a graphical guide of the main steps followed by GRECOSAR: 1) Snapshot of the blueprint of a vessel (a left) and the resulting meshed CAD model (a right), 2) snapshot of the bitmap generated by GRECO (b), 3) snapshot of the magnitude of the synthesized raw data (c) and final POLInSAR dataset (d). For GRECO bitmap, the colors indicate the sense of the normal vector of each surface according to the RGB code with red pointing to horizontal, green to the observer and blue to the vertical.

The main steps of a complete simulation from target modeling to simulated image and later analysis can be gathered in six main groups. They are detailed in the following, with the support of the snapshots of Fig. 3 that exemplify the most significant intermediate results.

Target modeling. This stage is devoted to manually build a parametric version of the model from hard or digital blueprints. Once finished, the parametric geometry is discretized into planar facets by the commented tessellation procedure. Several tests have shown that a **facet length** of **1 cm** provides an efficient trade-off between the degree of detail and computational efforts. The total number of facets in a model depends, besides facet length, in the chordal error, which fixes the minimum distance between a curved surface and the planar one discretizing it. For ship models around 70 m long, more than 4 · 10^5^ facets are managed with a **chordal error below 3 mm.**
Pre-Processing. This stage simulates the sensor point of view and the transmitted chirp signal. For the former, the platform path (both orbital or airborne), antenna pointing and target environment (location, orientation and dynamics) are considered. The result is a file defining the radar aspect angle at each synthetic aperture position via two *view* angles.^†^ For the latter, the chirp signal is simulated at base band in time domain. As GRECO^®^ works in the frequency domain, the chirp frequency samples are computed, and the related amplitude and phase terms of the chirp spectrum stored in a file for raw data synthesizing.
GRECO. This stage corresponds to EM simulation. According to the user-defined radar aspect angle, GRECO estimates the mono-static polarimetric EM field scattered by the input geometry for each of the frequency samples related to the chirp signal. It provides the value normalized to the incident field and takes both far- and near-field regimes according to the scenario configuration. In order to make this step faster, GRECO^®^ applies an additional discretizing step consisting on generating a bitmap of the input meshed target. The PC graphic workstation performs all the intense operation tasks and generates a bitmap image of the visible entities according to a user-defined **pixel size** value. Several tests have shown that a value of **1 cm** provides an efficient tradeoff between model realism and processing time, despite in some cases lower values are mandatory (specially when sea surface is adopted in the scene).
Post-Processing. This stage synthesizes the raw data in time domain from the contribution of the different magnitude and phase terms at each frequency. The complete SAR signal as received at the antenna is emulated by properly adding the complex chirp spectrum samples, the magnitude and phase terms due to target scattering (GRECO^®^ EM fields), the phase term due to the two-way signal propagation and the azimuth-dependent phase terms due to Range Cell Migration. The resulting raw data is windowed in the Doppler domain according to the azimuth antenna radiation pattern. In GRECOSAR, the temporal window is fixed by the time extend in which the signal impinges the target and, hence, the antenna radiation pattern can be assumed constant along range due to the reduced dimension of the scene.
SAR Processing. This stage focuses the raw data as done for real images. An efficient and platform-independent code of the Extended Chirp Scaling Algorithm (ECSA) is used [24].
Data Interpretation. GRECOSAR provides some utilities for data interpretation as CTD polari-metric processing, 3D image formation or zero-padding interpolation for analysis purposes.

Note that the numbers provided by the previous discretizing parameters (facet length, chordal error and pixel size) are A dependent and are valid up to **X band.**

## 3. Scattering Study

**Figure 4.**
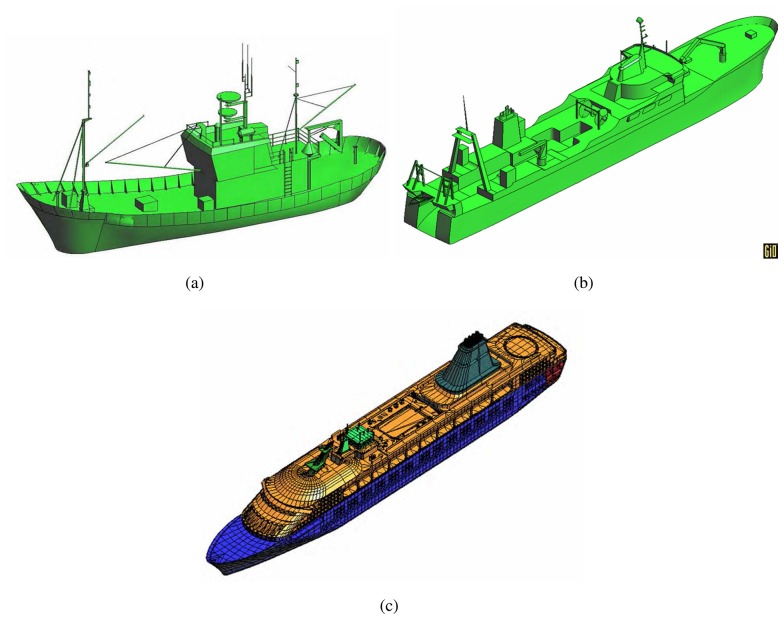
Snapshots of the SPA (a), ICE (b) and FER (c) models used in this work.

**Figure 5.**
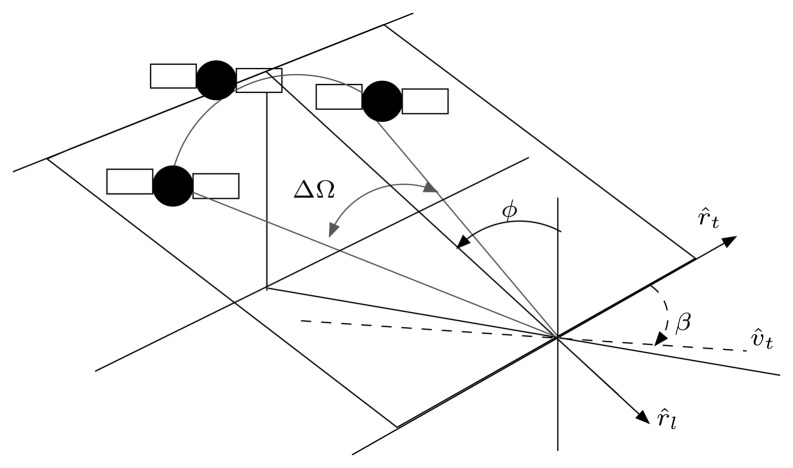
ISAR imaging geometry of GRECOSAR.

The main activity carried out with GRECOSAR has been focused to derive vessel scattering maps for diverse observation conditions [25]. Three different geometries have been considered (see Fig. 4), namely: 1) a Spanish fishing vessel (SPA) 27 m long and 10 m wide, 2) an Icelandic fishing vessel (ICE) 70 m long and 12 m wide and 3) a common passenger ferry (FER) 200 m long and 30 m wide. They have been processed in the Inverse SAR (ISAR) imaging geometry of Fig. 5 for a signal bandwidth of 1 GHz and angular aperture ΔΩ of 5 degrees. This imaging geometry is a type of circular spotlight mode for which image resolutions close to centimeters can be reached. In order to make image interpretation easier, those phenomena degrading the quality of images have been discarded. Specifically, neither ship motions nor sea clutter have been considered as they can cause undesired blurring effects [26, 27].

**Figure 6.**
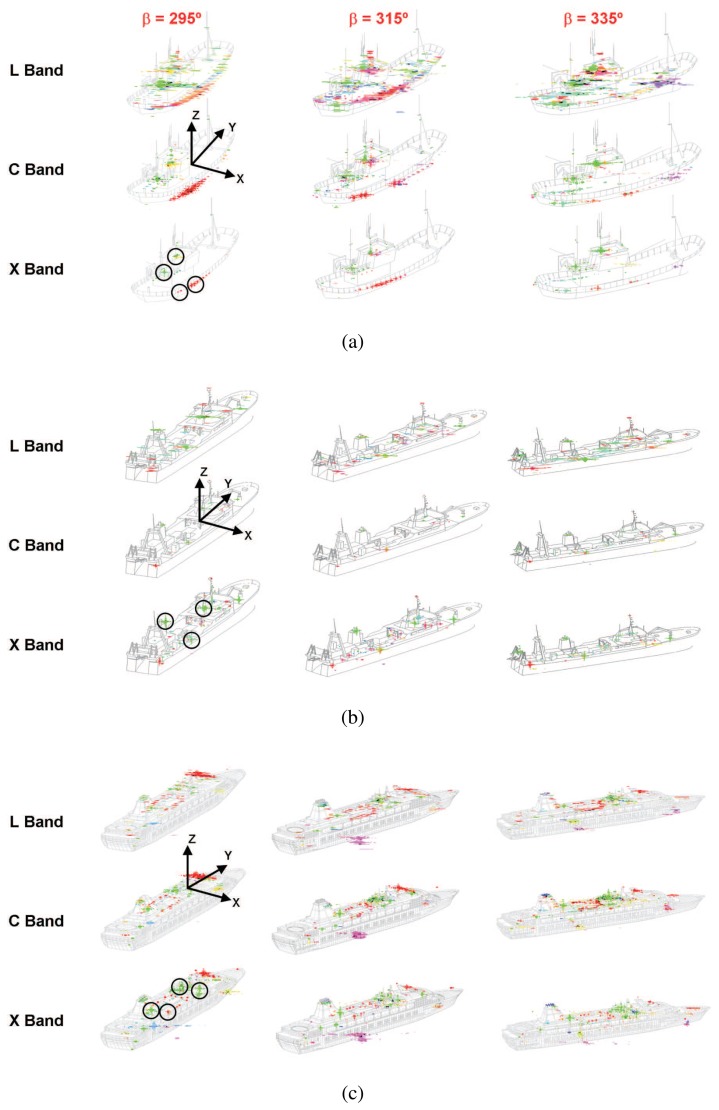
Scattering maps obtained for the SPA (a), ICE (b) and FER (c) vessels at L, C and X band with *β* ∈ ]295, 315, 335}° and *ϕ* = 20°. They have been analyzed with the Pauli CTD theorem for a dynamic range of 25 dB (red → 1*^st^* channel, green → 2*^nd^* channel, blue → 3*^rd^* channel). The lengths of the SPA, ICE and FER ships are 30, 70 and 200 meters. Circles highlight *PePS* (stable dispersors) with which the feature sets of Table 1 are defined.

The three models have been processed at L, S, C and X bands for seven bearings ranging from 295° to 355° in steps of 10°. All the derived images are fully-polarimetric and have been analyzed with the Pauli, SDH [5] and Cameron [6] CTD theorems. In the paper, only some cases analyzed with the Pauli theorem are provided as the results obtained with the remaining images and CTD theorems are very similar. Particularly, Fig. 6 presents the scattering maps retrieved at X, C and L bands for an incidence angle of *ϕ* = 20° and three different target orientations, *β* = ]295, 315, 335}°. The images depict the normalized Pauli significance with a RGB-based color code (red for the first Pauli mechanism -trihedral, sphere, flat plane,…-, green for the second -dihedral,…- and blue for the third -anti-symmetric mechanisms-) over a transparent snapshot of the vessel under the sensor point of view. A local coordinate system is also included to support PePS location, which are highlighted with the black circles.

With the previous scenario configuration, ISAR scattering maps become an important aid for improving the interpretation of SAR images. They allow to analyze the scattering properties within the different pixels making the isolation of small-scale details easier. As a result, the relation between ship geometry and the measured scattering may be properly defined.

The images of Fig. 6 show that each type of geometry presents a particular distribution of strong scatters, *PePS* candidates, with a high RCS and a well-defined polarimetric pattern that remains stable within a specific range of views. They mainly correspond to dihedral interactions of cylindric structures (like masts, funnels, …) and trihedral behaviors due to corner geometries (like buttresses, …). Two important issues arise from these results [25]: 1) the mechanisms of the main scattering centers are present in all the CTD bases and this makes the polarimetric interpretation to be independent of the selected theorem [28]; and 2) such mechanisms are related to scatterers with large electrical dimensions (around 1 meter for the analyzed geometries), which may explain an apparent scattering stability along frequency and the high RCS values observed in simulated scenes [2]. This asseveration is however preliminary and should be corroborated with real images when available. Certainly, real targets have more interfering elements (not simulated due to computational limitations) that could amend previous conclusions.

## 4. PePS-based feature set

The previous section has shown that the meso-scale characteristics of ships could be described by a particular distribution of intense and strongly-polarized scatterers that may make possible the discrimination of different geometries. This section makes a step further and evaluates the possibility to exploit such information for building a *feature set* useful for ship classification.

In order to fix an unequivocally way for isolating PePS with measurable parameters, a definition is required. According to the experience gained with the scattering studies, PePS can be considered those scatterers presenting a RCS 10 dB higher than the surrounding scatterers and a stable polarimetric behavior within a solid angle of at least π/3 steradian ^‡^[8]. Three issues are important in this definition: 1) stable polarimetric behavior for *PePS n* means to take the same value of $p_{n}^{j}$ within the selected aspect angle (see Equations 4 and 5); 2) *PePS* 3D positions, (*x_n_*, *y_n_*, *z_n_*), are expressed with respect to a local coordinate system defined at the target and 3) the threshold in the angular aperture is selected by the response of typical canonical scatterers, like trihedrals and dihedrals. This implies that PePS locate those structures with the closest scattering behavior to canonical targets.

**Figure 7.**
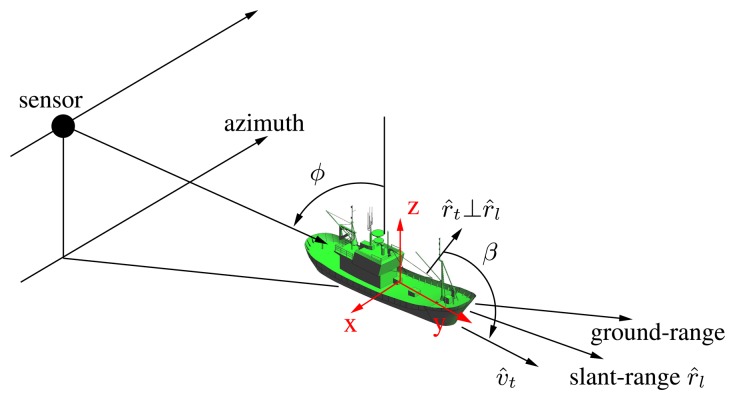
Local coordinate system used to define PePS within targets. The center is located at the center of mass.

With the previous definition, the PePS-based feature set can be mathematically formulated by [8]

(2) $$\Theta^{j}=\{\Theta_{n}^{j}\}\;\text{for}\;1<n<N_{\text{PePs}}$$

where *N_PePS_* indicates the number of PePS used to describe the geometry of target *j* and

(3) $$\Theta_{n}^{j}={\left\{x_{n},y_{n}.z_{n},p_{n}\right\}}^{j}$$

gathers the tri-dimensional location and polarimetric behavior of each guide scatter. On the one hand, location information is provided according to the local coordinate system defined in Fig. 7, which is centered at the center of mass of the target. On the other hand, polarimetric properties are provided by means of the Pauli mechanisms defined for the scatter *n* as

(4) $$\begin{array}{ccc} \text{Trihedral}\;<p_{n}^{j}>=0 & if & \left|p_{n,0}^{j}|>|p_{n,1}^{j}|,|p_{n,2}^{j}\right|\\ \text{Dihedral}\;<p_{n}^{j}>=1 & if & \left|p_{n,1}^{j}|>|p_{n,0}^{j}|,|p_{n,2}^{j}\right|\\ \text{Anti}-\text{symetric}\;<p_{n}^{j}>=2 & if & \left|p_{n,2}^{j}|>|p_{n,0}^{j}|,|p_{n,0}^{j}\right| \end{array}$$

where $\left|p_{n,xx}^{j}|=p_{n,xx}^{j}/\text{max}\{p_{n,0}^{j},p_{n,1}^{j},p_{n,2}^{j}\right\}$ define the normalized weight with

(5) $$\begin{array}{c} p_{n,0}^{j}=\frac{1}{\sqrt{2}}\frac{S_{hh}^{n}+S_{vv}^{n}}{2}\\ p_{n,1}^{j}=\frac{1}{\sqrt{2}}\frac{S_{hh}^{n}-S_{vv}^{n}}{2}\\ p_{n,2}^{j}=\frac{1}{\sqrt{2}}S_{hv}^{n} \end{array}$$

being

(6) $${\left[S\right]}^{n}={\left[\begin{array}{cc} S_{hh} & S_{hv}\\ S_{hv} & S_{vv} \end{array}\right]}^{n}$$

the mono-static master scattering matrix. Note that the usage of a reference positioning system different to the SAR azimuth/slant-range one needs from incidence and sensor-to-target relative orientation angles in order to make the coordinate system transformation. The former is derived from system design whereas the latter from any of the currently available methods for estimating vessel bearings [9, 29].

The previous selection procedure of PePS and the formulae related to the feature set have been applied to the dataset presented in Section 3.. The results show that each input geometry presents a particular and different reference feature vector [8]. All of them are summarized in Table 1. They are valid for the range of simulated observation conditions fixed by Φ*^SO^*^1^ = ]10 – 30°, 280 – 350°, 5.3 – 9.65*GHz*}*^SO^*^1^ that cover different range of values for incidence, target orientation and operating frequency. The margin of validity in terms of incidence angle and target orientation is notably large and open the door for characterizing a vessel with a reduced set of feature vectors.

In consequence, it appears that ship geometry may become characterized by a set of quantitative parameters, which are relatively easy to be measured with real SAR data. Certainly, the polarimetric terms $p_{n}^{j}$ are directly derived by a simple combination of the complex values retrieved for pixel *n* in all the polarimetric channels whereas the height (z coordinate) from interferometric measurements by [8]

(7) $$<Z_{n}^{j}>=\sin\phi \left[\frac{cr_{o}}{4\pi fB^{⊥}}\Delta \psi -\frac{\Delta r}{\tan\phi }\right]$$

where Δ*ψ* and *Δr* fix the interferometric phase and slant-range difference of scatter *n* with respect to a fixed reference. *r_o_* is the range and *B^┴^* the perpendicular baseline, both known radar parameters [30, 31]. The other two coordinates are obtained by geometrical manipulation

(8) $$<{\left[\begin{array}{c} x\\ y \end{array}\right]}^{n}>=\left[\begin{array}{cc} \cos<\beta > & \sin<\beta >\\ -\sin<\beta > & \cos<\beta > \end{array}\right]\cdot {\left[\begin{array}{c} sr/(\sin\phi )\\ \text{azi} \end{array}\right]}^{n}$$

where *azi* and *sr* indicates respectively the azimuth and slant-range position of scatter *n* with respect to the center of the ship signature. This point is estimated approximately as the central point of the linear feature generated by the ship signature and identified by the Radon transform. This image transformation also helps on estimating ship bearing (< *β* >) [9].

## 5. Vessel Classification Algorithm

VCA algorithm consists basically on estimating, from POLInSAR data, possible feature sets accord­ing to the formulae of previous section. The resulting vectors are then correlated with the reference ones and a similarity value is provided. The geometry which reference set provides the highest similarity is used to describe the observed target. The number of reference feature vectors (or patterns) should ac­complish a trade-off between geometry description accuracy and method robustness. On the one hand, a low number of vectors could lead to an overestimation of the classification rate providing a wrong knowledge about the real performance expected for VCA. On the other hand, an excessive number of patterns would make quite similar geometries to be classified in different classes. In this case, little modifications of target environment may generate unpredictable results and, hence, the same ship would not be classified with the same class.

For retrieving estimates of the feature vector, VCA analyzes the input POLInSAR data set with the quad-pol Pauli vector.^§^. This leads to three different interferograms (one per each Pauli mechanism) from which local maxima are isolated. All these *m* maxima are combined with the *M* PePS associated with pattern *p* generating $P=m\cdot \frac{M!}{(M-m)!}$ estimated feature vectors [7]. For the permutation 1 < *κ* < *P*, the similarity parameter $0<S_{p}^{\kappa }<1$ is [7, 8]

(9) $$S_{p}^{\kappa }\dot{=}\frac{r}{M}\cdot (1-\sum_{j=1}^{4}e_{j}^{p}\cdot W_{j})$$

where $0\geq e_{j}^{p}\geq 1$

(10) $$e_{j}^{p}=\frac{1}{r}\sum_{n=1}^{r}e_{j,n}^{p}$$

are the four errors related to the four elements of the PePS vector (*x*, *y* and *z* coordinate plus *p* polarimetric mechanism). In both formulas, *r* indicates the number of PePS with a corresponding scatter in the image and helps to better discriminate those patterns with no relation with the measured structure. In this sense, each permutation applies a *suitability scatter* step that discards, in the comparison between an estimated and reference feature set, those local maxima that induce a value of 1 in at least two of the four errors of Equation 9.

For *x* (*j_n_* = *x_n_*) and *y* (*j_n_* = *y_n_*) coordinates, the components error of Equation 10 are

(11) $$\begin{array}{ccc} e_{j,n}^{p}=1 & if & \frac{\left|<j_{n}>-j_{n}\right|}{\Delta j}\geq 1\\ e_{j,n}^{p}=0 & if & \frac{\left|<j_{n}>-j_{n}\right|}{\Delta j}<1 \end{array}$$

where *j_n_* indicates the coordinate of the selected dimension for the position of PePS n. < · > is the expectation operator and indicates the value of such coordinates for the scatter that has been associated with the PePS spot. Δ*j* is the image cell extend of the slant-range (for the *x* coordinate) and azimuth (for the *y* coordinate) dimension (see Equation 8). It gives a measure of the uncertainty on estimating the proper coordinates of PePS and, so, it is used to ponder the difference error. For the *z* coordinate

(12) $$\begin{array}{ccc} e_{z,n}^{p}=1 & if & \frac{\left|<z_{n}>-z_{n}\right|}{\Delta z}\geq 1\\ e_{z,n}^{p}=\frac{\left|<z_{n}>-z_{n}\right|}{\Delta z} & if & \frac{\left|<z_{n}>-z_{n}\right|}{\Delta z}<1 \end{array}$$

where *z_n_* plays the same role than *j_n_* in Equation 11 and Δ*z* corresponds to the height bias experi­mented by the system for a particular phase error (*σ_ϕ_*)

(13) $$\Delta z=\frac{\lambda \sin\phi r_{o}}{4\pi B_{⊥}}\sigma_{\phi }$$

Finally, for the polarimetric error

(14) $$\begin{array}{ccc} e_{p,n}^{p}=1 & if & <p_{n}>\neq p_{n}\\ e_{p,n}^{p}=0 & if & <p_{n}>\neq p_{n} \end{array}$$

where *p_n_* is the weight of the normalized Pauli significance as defined in Equations 4 and 5. In Equation 9, the factors (0 ≥ *W_j_* ≥ 1 for *j* ∈ [1 … 4]) are weights that give different “significance” to each error in the identification process. Empirical analysis have shown that the following values provide the best overall results (*W_x_* = 0.15 *W_y_* = 0.15 *W_z_* = 0.35 *W_p_* = 0.35). They reduce the influence of azimuth and range errors due to the image distortions induced by sea surface [1, 32].

In summary, the main steps of VCA are [2]:

1. Coregistrate single-pass interferometric pairs and generate Pauli interferograms.
2. Isolate all the local maxima (*m*) present in all the three Pauli interferograms for a fixed dynamic range (*DR_i_*) and find the values of the four components defining the feature set. In this step, a ship bearing estimation methodology based on the Radon transform has been used for making the required coordinate system transformation (see Section 4. Equation 8).
3. For each pattern with *M* PePS, test the similarity of the $m\cdot \frac{M!}{(M-m)!}$ permutations possible. Here, the formulation from Equation 9 to 14 applies with the *suitability scatter* step. This implies that, for a permutation *κ*, *r* could be lower than *M* if exists one or more local maxima that present, in at least two of the four components of the feature set vector, errors equal to 1.
4. That pattern with that permutation providing the highest similarity value is selected to classify the observed ship for the dynamic range *DR_i_*.
5. Repeat steps 2-4 for different dynamic ranges in order to reach the maximum similarity and/or the best discrimination among the different models. The model which feature set has been selected the highest number of times to classify the observed ship becomes the final decision of the algorithm. The final similarity value is the value providing the best discrimination among models.

Note that this rule is empirical and it has been motivated by the information that the algorithm has to deal with. It has been adopted because it allows to evaluate the performances of the proposed method in an easy and quick way. Obviously, better decision rules may be developed in the future.

## 6. VCA Performance evaluation

VCA performance has been evaluated with a set of simulations carried out at X band for the sensor summarized in Table 2. It deals with a standard single-pass interferometric configuration with an orthogonal baseline of *B^┴^* = 30*m*, height sensitivity of Δ*z* = 0.27*σ_ϕ_* (for a phase error expressed in degrees) and image resolution of 2.3 m in azimuth and 1.3 m in range [7]. By now, tests with real data have not been possible (despite they are mandatory for a proper validation of the technique) because no dataset with the required specifications ^¶^was available at the time this manuscript has been written. Future joint projects should consider new dedicated measurement campaigns where this and other methods can be tested and cross-correlated.

The SPA, ICE and FER geometries have been processed in four different scenarios with the reference feature sets listed in Table 1. In scenario 0 neither sea surface nor ship motions are included whereas in scenario 1 only ship motions, in scenario 2 only sea surface and in scenario 3 both ship motions and sea surface. On the one hand, ship motions are mainly described by rolling and pitching with the first-derivative angular velocities fixed in Table 3. They are defined counter-clockwise with respect to the rotation axis. On the other hand, sea surface is modelled with the two-scale height profile for *h_l_*=1.5 m, *h_s_*=0.1 m, *λ_l_*=100 m, *λ_s_* = *λ*/(2 sin *ϕ*) [33]. This last formula assures a Bragg scattering phenomena in the scene, which is sensitive to the operating wavelength [22].

**Figure 8.**
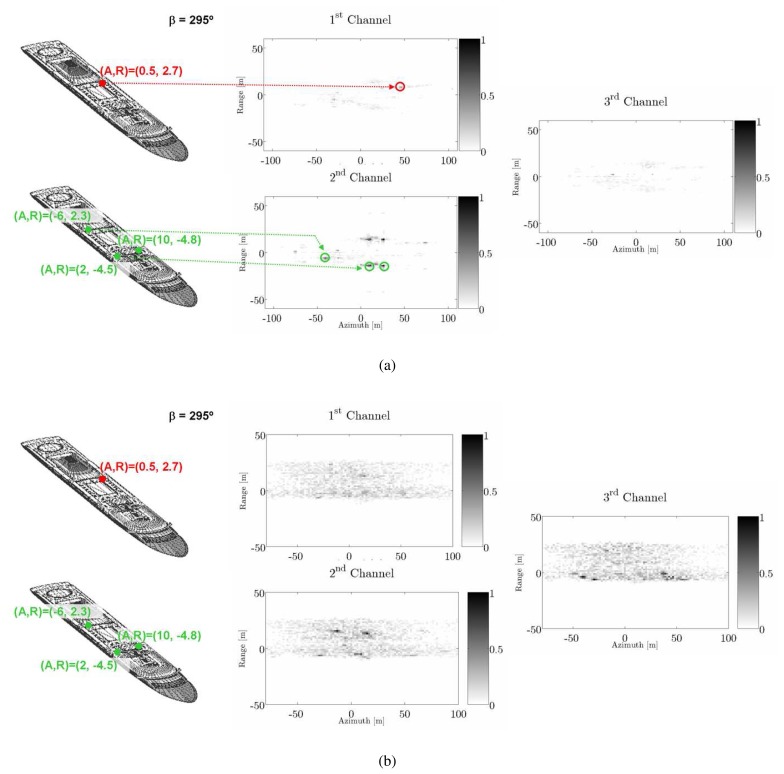
Magnitudes of the Pauli interferograms normalized to the overall maximum ob­tained for the FER model with *β* = 295° and ϕ = 20° in scenario 1 (a) and 3 (b;. Both images present a snapshot of the point of view with transparent surfaces. They have su­perimposed colored points that locate the azimuth x slant-range positions of the four PePS defining the feature set of FER ship, as stated in Table 1. Note how the presence of the sea distorts the ship signature making PePS isolation by simple eye inspection (colored arrows) more difficult.

Table 4 provides the similarity values calculated for each reference feature vector (*S_Θ^SPA^_ S_Θ^ICE^_ S_Θ__^FER^_*) when the three ships are processed in the four scenarios. As observed, ships are well classified in almost all situations preserving a reasonable confidence even with clutter. Two items are important, namely: 1) non-uniform azimuth shifts help in some cases to improve classification and 2) sea clutter appears to be the most adverse factor. The latter is specially adverse for the ICE model as the lack of PePS in the first Pauli channel makes the presence of the sea, with dominating sphere-like behaviors, to increase the confusion with respect to SPA and FER models. This can be appreciated in Fig. 8 where the snapshot of the dB magnitude of the three Pauli interferograms derived in scenario 1 and 3 for the FER ship and *β* = 295° are shown. The clutter modifies notably the scattering of the scene adding new trihedral-like mechanisms that can reduce the coherence and, thus, the quality of the retrieved heights [34]. Also interesting is the increased significance of the third channel due to the anti-symmetric mechanisms induced by sea, as happens in real life.

Therefore, it appears that PePS isolation is possible and needs from the information diversity provided by polarimetry and interferometry [2]. The practical benefits that this issue gives to ship classification are highlighted when single-polarized interferometric pairs are processed. This is observed in Table 5 where the similarities obtained for the ideal scenario 0 have been reprocessed with only the 3D location information within the HH channel (discarding, thus, the parameter *p_n_*). The test show that ship classification is not possible as the discrimination capability is notably reduced with the absence of polarimetry. Something similar happens for additional tests performed for a reduced baseline value of 20 m. In that case, classification confidence is sensitively reduced making ship classification fairly difficult [2].

## 7. Requirements for Real Scenarios

In the previous section, the analysis of simulated data has shown that VCA may be reliable for classifying ships in real scenarios. But two points are important before asseverating this, namely: 1) real imagery is mandatory in order to validate the results presented here, based on simulated scenarios and 2) sensor requirements may be too restrictive. Certainly, VCA tests in GRECOSAR have shown that single-pass POLInSAR sensors with recommended resolutions lower than 3 m should be adopted with effective baselines of at least 30 m long. This baseline value could be too long for spacecrafts where the presence of a large mast could introduce technical limitations, as shown in the NASA/JPL/DLR SRTM mission [35]. Incidence angles should be low so that the influence of the strong dihedral-like mechanisms that can appear at the lateral hull becomes less noticeable.

Regarding polarimetry, the usage of quad-pol modes adds certain operability limitations. Among them, the timing scheme is largely important as at least two pulses, one per each polarization, should be emitted in the slot time of one synthetic aperture position. This makes the system PRF to be two times the PRF of the images, strongly constraining the maximum swath and image resolution. A solution may lay on compact polarimetry for which specific polarimetric descriptors can be reconstructed without the necessity of quad-pol modes [36, 37]. In VCA context, the option of circular dual-pol (CC) schemes (emitting in right- or left-handed circular polarizations and receiving in both) have been evaluated [33]. Its main advantage lies on the possibility to distinguish with one pulse trihedral- and dihedral-like mechanisms, which actually are the mechanisms dominating the behaviors of the guide scatterers. Some tests have been performed for this mode and the results show a poor classification performance when the clutter becomes intense [38].

An alternative to skip the limitations of working with orbital POLInSAR data are the airborne platforms. Examples can be the F-SAR concept of DLR [39] or the RAMSES system of ONERA. The usage of airborne systems is not exempt, however, of additional limitations, like the limited coverage. Normal swath values does not pass from 10 Km, which may not be sufficient for covering large open sea areas. In addition, integration time is larger for airborne platforms making the sensor to be more sensitive to ship and sea dynamics. This makes the quality of the interferometric phase to be reduced. Ways to overcome this drawback may be to take profit of sub-aperture theory in order to process a specific portion of the spectrum where the scattering of the scene has not been notably changed [40, 41]. But this solution implies a reduction of image resolution depending on the sub-aperture, which in some cases may put PePS isolation at risk.

## 8. Conclusions

This paper has presented a complete overview of the research line carried out by UPC in the field of ship scattering characterization and classification. There, the development of a complete and realistic SAR simulation tool of complex targets (GRECOSAR) has been essential. First of all, it has allowed to relate the polarimetric scattering maps with the geometry of ships with a flexibility and control not possible in real scenarios. The derived conclusions regarding ship scattering, which have introduced the concept of PePS, have leaded to the definition of the *feature sets* useful for classification. In a second term, the availability of simulated data obtained within realistic scenarios has made possible to evaluate the performance of the VCA classification algorithm. There, high classification ratios are found even under the presence of strong sea clutter. In this sense, the gained experience has shown that VCA has a simpler decision rule (only the comparison of the similarity value) with respect to the other solutions that increases its robustness.

Regarding practical issues, VCA would permit to take profit of the data from the new second generation of orbital SAR sensors, such as TerraSAR-X, RADARSAT-2 or Cosmo-Skymed. The increased resolution and revisiting time seems to be suitable for the application specifications for which VCA has been thought, except they are not able to provide single-pass interferometric data. The future TanDEM-X mission can be an excellent opportunity for validating the performance of VCA with single-pass POLIn-SAR real data. But this would be only fruitful if measurement campaigns involving different institutions are promoted. Exploitation of airborne sensors may be also useful for validation purposes in spite of the limitations induced by the longer integration time, which makes the image more sensitive to vessel motions.

## Figures and Tables

**Table 1.** Feature vectors for targets *j* = *SPA*, *j* = *ICE* and *j* = *FER* and Φ*^SO^*^1^ = ]10 – 30°, 280 – 350°, 5.3 – 9.65*GHz*}*^SO^*^1^. Heights (z) are normalized with respect to the lowest value in order to match interferometric height conventions.

Θ*^SPA^*					Θ*^ICE^*				

$\Theta_{i}^{\text{SPA}}$	*x_i_*	*y_i_*	*z_i_*	*p_i_*	$\Theta_{i}^{\text{ICE}}$	*x_i_*	*y_i_*	*h_i_*	*p_i_*

1	3.5	-10	0	0	1	3	-2	0	1
2	3.5	-5	0	0	2	-6	-14	6.5	1
3	-0.5	-8	2.5	1	3	-1	1	6.5	1
4	-0.5	-2	4.5	1	-	-	-	-	-

Θ*^FER^*					-				

$\Theta_{i}^{\text{FER}}$	*x_i_*	*y_i_*	*z_i_*	*p_i_*	-				

1	12	-25	0	0	-				
2	10	-25	0.5	1	-				
3	9	8	2.5	1	-				
4	9	-8	2.5	1	-				

**Table 2.** The X-band SAR sensor based on DLR's TerraSAR-X to test VCA performance.

*ϕ* [°]	*20*	*r_o_* [*Km*]	*550*	PRF [Hz]	*3630*
f [GHz]	*9.65*	BW [MHz]	*135*	*τ*[*μs*]	*25*

**Table 3.** Scenario configurations for simulations in Section 6.. Sea parameters are *h_l_* = 1.5 m, *h_s_* = 0.1 m, *λ_l_*=100 m, *λ_s_* = *λ*/(2 sin ϕ. Rotational motions are expressed with first-order angular velocities in rad/s

Scenario	Bearings	Motions	Sea surface	*β*	*δ̇_roll_*	*δ̇pitch*	*β*	*δ̇_roll_*	*δ̇_pitch_*
0	[295:10:355]°	No	No	295	*-1.56*	*-0.26*	335	*-0.98*	*-1.16*
1	[295:10:355]°	Right Side	No	305	*-1.43*	*-0.52*	345	*-0.76*	*-1.32*
2	[295:10:355]°	No	2 scale	315	*-1.32*	*-0.76*	355	*-0.52*	*-1.43*
3	[295:10:355]°	Right Side	2 scale	325	*-1.16*	*-0.98*	-	-	-

**Table 4.** Similarity values 0< S < 1 retrieved for X band simulations in scenarios 0 (up-left), 1 (up-right), 2(down-left) and 3 (down-right) with the quad-pol mode. Bold numbers indicate the model classifying the processed geometry, so if arranged along the matrix diagonal, VCA performs good.

*β* = 295° | *β* = 315°	*Θ^SPA^*	*Θ^ICE^*	*Θ^FER^*	*β* = 295° | *β* = 315°	*Θ^SPA^*	*Θ^ICE^*	*Θ^FER^*
Processing SPA	**0.82** | **0.8**	0.31 | 0.47	0.3 | 0.32	Processing SPA	**0.76** | **0.71**	0.22 | 0.11	0.22 | 0.4
Processing ICE	0.08 | 0.0	**0.56** | **0.82**	0.08 | 0.28	Processing ICE	0.0 | 0.32	**0.7** | **0.76**	0.08 | 0.45
Processing FER	0.1 | 0.11	0.19 | 0.0	**0.7** | **0.6**	Processing FER	0.34 | 0.1	0.26 | 0.0	**0.74** | **0.76**
*β* = 295° | *β* = 315°	*Θ^SPA^*	*Θ^ICE^*	*Θ^FER^*	*β* = 295° | *β* = 315°	*Θ^SPA^*	*Θ^ICE^*	*Θ^FER^*
Processing SPA	**0.62** | **0.63**	0.26 | 0.22	0.31 | 0.21	Processing SPA	**0.57** | **0.44**	0.15 | 0.0	0.0 | 0.25
Processing ICE	0.0 | 0.17	**0.33** | **0.45**	0.0 | 0.08	Processing ICE	0.1 | **0.7**	**0.8** | 0.44	0.1 | 0.25
Processing FER	0.11 | 0.0	0.33 | 0.0	**0.5** | **0.57**	Processing FER	0.21 | 0.0	0.3 | 0.0	**0.69** | **0.56**

**Table 5.** Similarity values 0< *S_p_* < 1 retrieved for X band simulations in scenario 0 with the single-pol mode. Bold numbers indicate the model classifying the processed geometry, so if arranged along the matrix diagonal, VCA performs good.

*β* = 295° | *β* = 315°	SPA*_pat_*	ICE*_pat_*	FER*_pat_*
Processing SPA	0.0 | 0.0	**0.1** | **0.35**	0.0 | 0.0
Processing ICE	**0.38** | 0.0	0.0 | 0.0	0.0 | **0.21**
Processing FER	**0.1** | **0.13**	0.0 | 0.1	0.0 | 0.0

## Footnotes

* Small facets are selected for modeling sea surface in order to meet the criteria followed with ships and allow accurate sea-ship interaction evaluation.

† For interferometric simulations the slave image have the same information than the master one, but compensated by the baseline vector.

‡ This is the solid angle subtended by a cone of 30° of aperture.

§ Due to the Pauli decomposition is a complete representation, the same simple mechanisms are isolated in any basis, for instance linear or circular.

¶ Main specifications are accurate ground-truth, maritime scenario with different known vessels, fine resolution and fine radiometric calibration
